# Comparison of end tidal carbon monoxide (eCO) levels in shisha (water pipe) and cigarette smokers

**DOI:** 10.1186/1617-9625-12-10

**Published:** 2014-07-04

**Authors:** Saima Akhter, Usman Ali Warraich, Nadeem Rizvi, Nusrat Idrees, Fatima Zaina

**Affiliations:** 1 Post-graduate trainee in pulmonology Department, Jinnah Post Graduate Medical Center, Karachi, Pakistan; 2 Chair Person business administration, Indus University, Karachi, Pakistan; 3 Head of Department in pulmonology Department, Jinnah Post Graduate Medical Center, Karachi, Pakistan

**Keywords:** Shisha, End tidal carbon monoxide levels (eCO), Cigarette smoker, Shisha smokers, Shisha cafes, Restaurants

## Abstract

**Background:**

Measuring eCo is rapid, non-invasive and inexpensive tool and correlate correctly with carboxyhemoglobin levels in blood. The aim of this study was to evaluate and compare the increase in end tidal carbon monoxide (eCO) levels in exhaled breath of passive smokers and healthy smokers after cigarette and shisha smoking.

**Findings:**

In a cross sectional study eCO levels were measured in 70 subjects (24 cigarette smokers, 20 shisha smoker, 26 passive smokers) by use of portable device. Smokers were asked to smoke shisha for 30 mins in shisha cafe or to smoke 5 cigarettes in 30 mins in a restaurant. eCo levels were measured at baseline (30 mins), 35 mins, 60 mins and 90 mins in all groups after entry to the venue. The baseline mean eCO level among cigarette smokers was 3.5 +/- 0.6 ppm (part per million), passive cigarette smokers 3.7+/-1.0 ppm, shisha smokers 27.7+/-4.9 ppm and passive shisha smokers 18.3+/-8.4 ppm .The mean increase in eCO after 90 min among smokers was 9.4+/-4.6 (p < 0.005), passive cigarette smokers 3.5+/-2.5 (p < 0.05), shisha smokers 57.9+/-27.4 (p <0.005) and passive shisha smokers 13.3+/-4.6 (p = 0.03).

**Conclusion:**

Exposure to shisha smoke is a cause of elevated eCO in smokers and passive smokers and due to in-door pollution, sitting in shisha bar causes significant increase in eCO levels.

## Findings

### Background

Water pipe is traditionally known as shisha, hukka, argileh, goza and hubble-bubble. Shisha smoking is more often considered as social activity that takes place at cafes, restaurants, parties and almost always practiced in groups [1] It has reported that the mean age of initiating shisha in Pakistan is 14 years and that the majority of youth prefer shisha smoking at shisha bars [2] where as a study in Saudi Arabia showed that 63.8% students start smoking shisha between the age of 16 to 18 and 70.2% of these individuals cite socialization as a primary reason for their shisha use [3].

Moreover, in Pakistan 78.5% of shisha smokers reported positive parental approval for this smoking habbit [1], while research done in 4 different universities of Pakistan indicated a shisha prevalence of 53.6% among students and factors most commonly identified for shisha smoking were curiosity(61.4%), pleasure-seeking (46.9%), peer pressure(22.8%), boredom(17.8%) and stress (10.8%) [1].

Measuring eCO (exhaled Carbon mono oxide) levels via smokerlyzer is an easy, rapid, inexpensive, non-invasive and convenient method [4]. eCO correlate correctly with carboxyhemoglobin levels in blood [5] and is an immediate useful tool to assess smoking habit of any person [4,6]. eCo in expired breath of person can also be used effectively as quantitative measure of passive smoking [6].

The aim of this study was to evaluate and compare the increase in end tidal carbon monoxide (eCO) levels in the exhaled breath of smokers after cigarette and shisha smoking. eCO levels were also analyzed in passive smokers.

## Methods

### Study design and participants

This was a cross-sectional study, which was done in restaurants of Karachi, Pakistan. A total of 11 venues were included and were grouped into 2 categories: Shisha cafes, exclusively designed for shisha smoking and restaurants, where shisha smoking was not allowed but cigarette smoking was allowed in dining area by the management. All venues selected for this study were closed indoor spaces with air-condition. Open- air restaurants exposed to outdoor sources of pollution (like a Bar-B-Q grill area and vehicle pollution) were excluded as were restaurants with an obvious source of indoor air pollution, such as communicating kitchen with dining areas.

70 volunteers, who visited these selected venues, were included in the study. 24 of them were cigarette smokers and 14 were passive cigarette smokers in the restaurants, 20 were shisha smokers and 12 were passive shisha smokers from shisha bars. Background information regarding their age, gender, occupation, smoking habits and illicit drug use including alcohol [7] was taken. People with a history of diabetes, asthma, COPD or having any other respiratory ailments were excluded [7,8]. The study population was divided into 4 groups. A cigarette smoker was defined as a person who smokes average of 5 cigarettes a day for at least last 3 years. Shisha smokers reported a smoking history of 3 years and who visit shisha cafes at least once a week for shisha smoking. Passive cigarette smokers and passive shisha smokers were that population who themselves had never smoked shisha or cigarette but they visited to these venues regularly.

The data was collected by team of doctors from Jinnah Post Graduate Medical Centre from October 2011 to December 2011.

During the pilot study on 18 volunteers, it was concluded that on an average a smoker took 5 minutes to smoke one cigarette and can smoke maximum of 5 cigarettes in 30 minutes therefore for comparable data the smokers were asked to smoke 5 cigarettes in 30 minutes in common restaurant or to smoke individual shisha for 30 minutes in Shisha bars. While sampling passive smokers were sitting on same tables where smoking was taken place. eCO levels in all 4 groups were measured at 30 minutes after arrival in venue and then at different intervals (35 minutes, 60 minutes and 90 minutes).

### Study ethics

Ethical permission was taken from Pakistan Chest Society and Jinnah Post Graduate Medical Center’s ethical committee. A formal permission from management of venues and informed consent from all volunteers was taken.

### Equipment and measurement variables

The equipment used for measuring end tidal Carbon monoxide level in exhaled air was MicroPlus Smokerlyzer (BedFont Instrument; UK). Previously it had been reported that smokerlyzer measurements of eCO correlate closely with blood carboxyhemoglobin concentration [5,6]. The smokerlyzer measures exhaled Carbon monoxide levels in part per million (ppm). A pre decided protocol was followed throughout the study to standardize all breaths being analyzed by device. The study subjects were asked to inhale deeply, hold their breath for at least 15 seconds, and then exhale rapidly and forcefully into disposable moth piece of instrument [9]. Single measurement was taken every time and maneuver was repeated only when the subject was unable to do it properly.

### Statistical analysis

Data entry and analysis was done by using Statistical Package for Social Sciences (SPSS) Version 19. Results were expressed in mean+/- SD.

The Welch was used instead of one-way Anova to compare mean of baseline eCO between all 4 groups because variance among all groups were not equal. Tamhane was used depending on sample distribution. Paired T Test was used for comparison between data from different intervals. A P value less than 0.05 was considered statistically significant.

## Results

**Figure 1:**
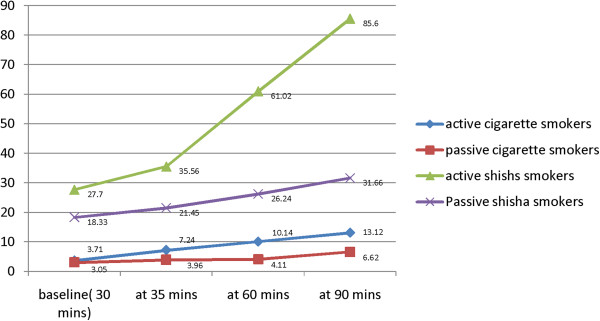
Mean end-tidal Carbon monoxide levels at different intervals after entering in venues (p <0.005).

The mean age of volunteers was 23.2+/-1.9. Ninety two percent of study population were students. Overall 68% were males and 32% were females. The baseline mean eCO value in cigarette smokers was 3.5 +/- 0.6 ppm; in passive cigarette smokers 3.7+/-1.0 ppm; in shisha smokers 27.7+/-4.9 ppm and in passive shisha smokers was 18.3+/-8.4 ppm. This baseline eCo levels were measured after 30 +/-5 minutes of subject’s entrance in these venues. Table 1.Mean eCO of all 4 groups was compared by use of the Tamhane test. Only the differences between baseline eCO levels of active and passive cigarette smokers was not significant (p = 0.985) but comparisons among other groups were highly significant (p <0.001). The difference between of eCo levels of active cigarette smokers and active shisha smokers was 24.2 ppm, active cigarette smokers and passive shisha smokers was 14.8 ppm, and between active shisha smokers and passive shisha smokers was 9.3 ppm Figure 1.

**Table 1:** Mean difference of eCO Levels among different groups 30 minutes after entering the venue

**(I) respondents (J) respondents**	**Mean difference of eCO (ppm) (I-J)**	**Std. error**	**Sig.**
Active shisha smokers	-24.20	1.10	<0.001
Passive shisha smokers	-14.83	2.43	<0.001
Active cigarette smokers	.21	.31	0.981
Active shisha smokers	-23.98	1.13	<0.001
Passive shisha smokers	-14.61	2.44	<0.001
Active cigarette smokers	24.20	1.10	<0.001
Passive cigarette smokers	23.98	1.13	<0.001
Passive shisha smokers	9.36	2.66	0.010
Active cigarette smokers	14.83	2.43	<0.001
Passive cigarette smokers	14.61	2.44	<0.001
Active shisha smokers	-9.36	2.66	0.010

eCo levels increased from baseline in all four groups after exposure to cigarette and shisha smoking. The difference in eCO (eCO 90 minutes –eCO 30 minutes) in cigarette smokers was 9.41+/-4.67 (p < 0.001), among passive cigarette smokers 3.57+/-2.53(p < 0.001), among shisha smokers 57.90+/-27.43 (p <0.001) and passive shisha smokers 13.33+/-4.69(p = 0.03).

## Discussion

Our study indicated that exposure to active or passive cigarette or shisha pipe smoking caused significant increase in eCO levels within indoor restaurants and shisha bars. The results of our study correlate well with few other studies done previously. In a study from India Sheetu Singh et al proved that eCO levels after hookah smoking were significantly higher than cigarette smoking therefore it cause substantial higher toxicity [10]. Eissenberg and Shihadeh from USA showed three times higher eCO levels in smokers after water-pipe smoking relative to cigarette smoking [11].

This centuries-old tradition of smoking is widely perceived to be less harmful than other forms of tobacco but shisha smoke contain higher concentration of carbon monoxide [11] and many other dangerous substances, including charcoal and heavy metals like arsenic, cobalt and is strongly associated with many adverse health consequences such as coronary artery disease, asthma exacerbation, COPD, infection diseases and lung cancer [12].

The main limitations should be noted was that this was a convenience sample study, and hence results may not be generalizable to the general population. We did not take measurements of eCO before entering the restaurants/cafes, and only performed eCO measurements between 30-90 minutes after entering the venue, hence factors that took place within these 30 minutes may have affected eCO levels, a factor we could not control for.

However, our study results showed significant raised baseline eCO levels in active and passive smokers in shisha cafes even before initiation of smoking and it was more likely because of exposure to indoor environment of venues for 30 minutes. Moreover, information of the number of cigarettes or shisha pipes smoked within the entire venue was not recorded. Further research is needed to take the above factors into account.

## Conclusion

Active and passive exposure to Shisha smoking is a cause of elevated eCO in smokers and non smokers and due to in-door pollution, sitting in shisha bar itself causes significant increase in eCO levels.

This study adds further support to existing evidences against Shisha smoking and can also be utilized as scientific basis for strict legislation on indoor shisha smoking in Pakistan as well as in other countries threatened by a growing rise in water pipe smoking.

## Competing interests

The authors declared that they have no competing interests.

## Authors’ contributions

SA was the lead author on the paper. UAW developed the research questionnaire and did the statistical analysis and helped in conducting the survey. NR supervised the project including manuscript preparation and submission. NI and FZ conducted the survey, assisted in manuscript writing. All authors read and approved the final manuscript.
